# Post-infectious irritable bowel syndrome after intercontinental travel: a prospective multicentre study

**DOI:** 10.1093/jtm/taad101

**Published:** 2023-07-31

**Authors:** Jiyang Chan, Niels van Best, Markia Ward, Maris S Arcilla, Jarne M van Hattem, Damian C Melles, Menno D de Jong, Constance Schultsz, Perry J J van Genderen, John Penders

**Affiliations:** School of Nutrition and Translational Research in Metabolism (NUTRIM), Department of Medical Microbiology, Infectious Diseases and Infection Prevention, Maastricht University Medical Centre, Maastricht, The Netherlands; School of Nutrition and Translational Research in Metabolism (NUTRIM), Department of Medical Microbiology, Infectious Diseases and Infection Prevention, Maastricht University Medical Centre, Maastricht, The Netherlands; Institute of Medical Microbiology, RWTH Aachen University Hospital, Aachen, Germany; School of Nutrition and Translational Research in Metabolism (NUTRIM), Department of Medical Microbiology, Infectious Diseases and Infection Prevention, Maastricht University Medical Centre, Maastricht, The Netherlands; Department of Medical Microbiology and Infectious Diseases, Erasmus University Medical Centre, Rotterdam, The Netherlands; Department of Medical Microbiology, Amsterdam University Medical Centres, Location AMC, University of Amsterdam, Amsterdam, The Netherlands; Department of Medical Microbiology and Medical Immunology, Meander Medical Centre, Amersfoort, The Netherlands; Department of Medical Microbiology, Amsterdam University Medical Centres, Location AMC, University of Amsterdam, Amsterdam, The Netherlands; Department of Medical Microbiology, Amsterdam University Medical Centres, Location AMC, University of Amsterdam, Amsterdam, The Netherlands; Department of Global Health, Amsterdam Institute for Global Health and Development (AIGHD), University of Amsterdam, Amsterdam UMC, Amsterdam, The Netherlands; The Institute for Tropical Diseases, Erasmus University Medical Centre, Rotterdam, The Netherlands; School of Nutrition and Translational Research in Metabolism (NUTRIM), Department of Medical Microbiology, Infectious Diseases and Infection Prevention, Maastricht University Medical Centre, Maastricht, The Netherlands; School for Public Health and Primary Care (Caphri), Maastricht University Medical Centre, Maastricht, The Netherlands

**Keywords:** Prospective, multicentre, post-infectious irritable bowel syndrome, Intercontinental travel

## Abstract

By longitudinally following a large cohort of intercontinental travellers, this study highlights the importance of considering multiple risk factors to comprehend post-infectious irritable bowel syndrome (PI-IBS). Stomach cramps, antibiotic use and nausea during travel were amongst the variables that predicted PI-IBS development following an episode of traveller’s diarrhoea.

Post-infectious irritable bowel syndrome (PI-IBS) can arise following acute infectious gastroenteritis. Despite increasing epidemiological understanding, its pathophysiology remains elusive. Understanding and preventing PI-IBS requires identifying modifiable risk factors and individuals at risk. The cross-sectional design of most previous study, however, prevents the estimation of the incidence of PI-IBS. Moreover, most studies inconsistently reported confounding adjustments, restricting the inference that can be drawn from their results.^1^ Given the strong association with traveller’s diarrhoea (TD), several previous cohorts have evaluated PI-IBS development amongst travellers.^2^ However, these studies investigated a limited number of predictors prior to or during the onset of PI-IBS. We therefore assessed the development of new-onset PI-IBS in association to an extensive set of potential predictive factors within the worldwide largest longitudinal traveller’s cohort.

As part of the COMBAT study,^3^ 2001 travellers were recruited from outpatient clinics in the Netherlands between 2012 and 2013. Questionnaires, including ROME III questions for functional gastrointestinal disorders,^4^ were filled before travel, immediately after return and at 1,3,6 and 12 months after return. Ethical approval was granted by the Medical Ethical Committee of Maastricht University Medical Centre (METC-12-4-093).

For the present study, we focussed on travellers at-risk for PI-IBS development, which was defined as travellers without IBS symptoms prior to travel and who experienced diarrhoea during travel (TD). TD was defined as ≥3 loose stools per day for at least 1 day. At-risk travellers with acute onset of IBS symptoms that continued for at least 6 months following the episode of gastroenteritis during their travel were considered PI-IBS cases (Figure 1A).

**Figure 1:**
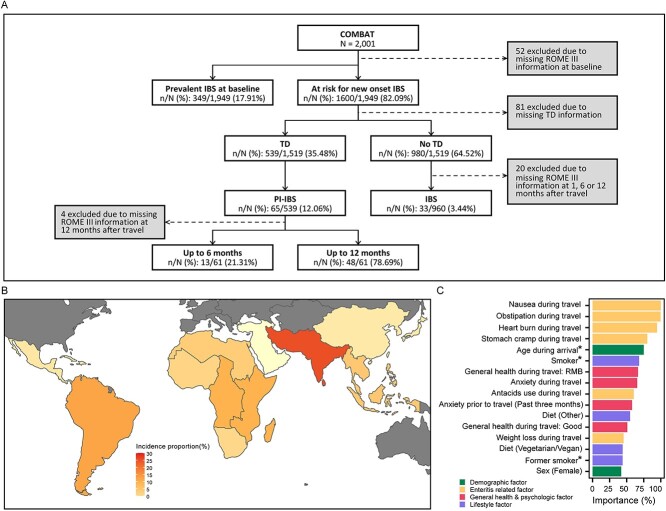
(A) Flowchart of study (B) Incidence proportion (%) of travellers that developed PI-IBS after TD per United Nations sub region. (C) Top 16 feature contribution for the neural network model displayed as importance in percentages from 0% to 100%. A neural network model is a machine learning method consisting of interconnected artificial neurons organized in layers that adjusts weights between neurons during model training to minimize errors. The importance of input variables on the network’s outcomes were calculated by analysing the weights and connections associated with each variable, as well as the changes in performance when these variables are altered or removed. RMB = reasonable, mediocre or bad: *inverse association with PI-IBS development.

Logistic regression models were used to identify predictors of PI-IBS development. Results were presented as odds ratios (OR) and 95% confidence intervals (95%CI). Subsequently, various machine learning (ML) approaches were used to predict PI-IBS onset in at-risk travellers. The best predictive model was chosen based on overall area under the receiver operating characteristic (AUROC) value. See Supplementary Data for a more detailed explanation of the methods.

A total of 33 out of 960 (3.4%) travellers developed IBS without TD after travel whilst 65 out of 539 at-risk travellers developed PI-IBS (relative risk: 3.51, 95%CI 2.34–5.26, *P* < 0.001). In addition, the majority (78.7%) retained PI-IBS symptoms up to 12 months after travel. The overall incidence of PI-IBS development after TD was 12.1% (95%CI 8.3–13.9). This high incidence agrees with the estimated overall point prevalence of PI-IBS after gastroenteritis of 11.5% in a large meta-analysis.^1^ However, the incidence was more than twice as high compared to another meta-analysis (pooled incidence of 5.4%) specifically focussing on PI-IBS after TD.^2^ This difference could be attributed to the limited size and high variability in study design between studies included in this latter meta-analysis.

Unique to our study was the ability to calculate incidence proportions according to travel destination. The highest incidence was observed in travellers to Southern Asia of which 14 out of 53 at-risk travellers developed PI-IBS (26.4%, 95%CI 12.2–27.5, Figure 1B, Supplementary Table 1). Geographic differences in the most prevailing causative agents of gastroenteritis are likely contributing to the variations in PI-IBS incidence proportions according to travel destination. Amongst gastroenteritis agents associated with PI-IBS, enterotoxigenic *Escherichia coli* is the predominant cause in South America, Africa and Southern Asia whilst *Campylobacter* is more common in Southern and South-East Asia.^1^^,^^5^

We subsequently examined risk factors for PI-IBS. Stomach cramps during travel were the strongest independent predictor for PI-IBS acquisition (OR_adjusted_ 5.92, 95%CI 2.17–16.12) (Supplementary Table 2). Pre-existing chronic illness (2.35, 95%CI 1.08–5.13), following a specific diet (vegetarian/vegan: 3.49, 95%CI 1.13–10.73; other diet: 5.69, 95%CI 1.56–20.82), reporting less than excellent general health during travel (good: 5.09, 95%CI 1.59–16.33; reasonable, mediocre or bad: 5.47, 95%CI 1.23–24.33), nausea during travel (2.25, 95%CI 1.08–4.69), sustained weight loss during travel (2.24, 95%CI 1.07–4.70) and taking antibiotics during travel (2.96, 95%CI 1.14–7.66) were also positively associated with PI-IBS development.

Thereafter, we applied ML models to predict PI-IBS development on complete cases (*N* = 510, 61 developed PI-IBS, Supplementary Figure 1). We subsequently proceeded with a neural network (NN)-model (weight decay = 4; hidden layer size = 5) as this algorithm performed slightly better than other models. The NN-model was able to classify new-onset PI-IBS with an overall AUROC of 0.77 (95%CI 0.75–0.79), median sensitivity of 0.66 (95% CI 0.63–0.70, bootstrap method) and specificity of 0.73 (95%CI 0.72–0.74). Like the multivariable regression, health issues related predictors during travel contributed most to the prediction of developing PI-IBS (Figure 1C).

Our study highlights the relevance of including multitudes of predictors prior to, during and after gastroenteritis to elucidate factors contributing to PI-IBS development. Several associations in our study coincided with observations found in previous studies, including antibiotic use and weight loss.^1^^,^^6^

Amongst the factors in the multivariable logistic regression; antibiotic use, nausea, stomach cramp and weight loss during travel were also found to be important predictors for PI-IBS development after TD in the NN-model. Along with TD, this may represent a more severe infection and subsequently a higher risk of developing PI-IBS. Other indicators of more severe infection were not statistically significantly increased (fever 7.9% vs 15.6%, *P* = 0.10) or reported too rarely to be included in the analyses (dysentery reported by 10 ‘at-risk’ travellers).

Some other associations with PI-IBS development after TD diminished during multivariable analysis. Sex and anxiety, previously identified as risk factors for PI-IBS,^1^^,^^6^ lost statistical significance in the multivariable analysis. This might be explained by the inclusion of several confounders and/or factors in the causal pathway of sex and PI-IBS. For example, whilst stomach cramps might reflect more severe gastroenteritis, it could also relate to sex hormone and/or anxiety-related differences in nociception or pain perception. Nevertheless, anxiety prior to travel and anxiety during travel were both important predictors in the NN-model. Anxiety is known to contribute to somatization.^7^ Moreover, anxiety may increase susceptibility to gastroenteritis, potentially due to stress-induced impaired barrier function or diminished immune function with onset of PI-IBS as conceivable consequence.^8^ In addition, smoking appeared to have a protective effect on the development of PI-IBS. Smoking has been recognized to improve ulcerative colitis, presumably through anti-inflammatory effects mediated by compounds in cigarette smoke. However, the precise mechanisms remain unknown.

Our prediction model revealed antacids use during travel as an important predictor of PI-IBS development. Antacids potentially enhances gastroenteritis severity by reducing stomach acid, a crucial barrier against infection, thereby increasing the risk of developing PI-IBS. Indeed, in a recent murine study, antacid pre-treatment expanded enteropathogen founding population size in the gut and heightened susceptibility to enteric infection with subsequent exacerbation in morbidity.^9^

Using Rome III, rather than Rome IV criteria to assess IBS might be considered a limitation of our study. However, Rome IV criteria are more restrictive and might underestimate the true incidence in epidemiological surveys.^10^ The lack of external data to validate the ML models might also be considered a limitation. However, this could not be tackled due to the unique character of our cohort.

Despite these potential limitations, this was, to our knowledge, the first study incorporating a comprehensive collection of predictors in prospectively investigating PI-IBS development after TD. Our results showed that the development of IBS symptoms after TD is substantial and strengthens our understanding of the multifactorial nature of PI-IBS.

## Supplementary Material

Supplementary_material_version5_07072023JC_unmarked_taad101
