# Supplementary material for: Post-infectious irritable bowel syndrome after intercontinental travel: a prospective multicentre study
Source: J Travel Med. 2023 Jul 31;30(6):taad101. doi: 10.1093/jtm/taad101 (PMC10628768; doi:10.1093/jtm/taad101)
Supplement: Supplementary_material_version5_07072023JC_unmarked_taad101 [file supplementary_material_version5_07072023jc_unmarked_taad101.docx]

**Supplementary material**

**Extended methods Logistic regression**

In order to mitigate unstable estimates and large standard errors, a selection method based on the approach proposed by Hosmer and Lemeshow was applied to identify predictors associated with the development of PI-IBS after travel.^1^ The process began by performing univariable analysis for each individual predictor as independent variable and PI-IBS development as dependent variable. Variables were then selected based on a cut-off point of p<0.25(Wald test).. Using the more conventional threshold of 0.05 may fail in identifying factors that are known to be important.^2^ Variables were tested for multi-collinearity before being entered into the multivariable analysis and machine learning process. In the multivariable analysis, statistically insignificant variables (p>0.05, Wald test) were removed one at a time. In case the removal of a variable resulted in a change in one of the remaining parameter estimates with >20% compared to full model, the variable was retained as it suggests confounding. The final model only included significant variables (p<0.05) and confounders. Statistical analyses were performed using IBM SPSS Statistics (version 27).

**Extended methods machine learning process**

Random forest (RF), neural networks (NN), and Support Vector Machine with Radial Basis Function kernel (SVM-RBF) were used to predict PI-IBS onset in at-risk travellers. In the machine learning process, only complete cases were considered and 5-fold cross-validation was repeated 10 times for performance assessment. This entails dividing the data into subsets, training the data on one subset, and employing the other subset to assess the model's performance. To account for variability, multiple iterations of cross-validation was performed utilizing distinct subsets derived from the same dataset. The validation outcomes from these iterations were next aggregated to derive an approximation of the model's predictive capability. Within the repeated cross-validation framework, hyperparameter tuning was performed using Grid Search combined with down sampling to achieve equal class distributions. Area under the receiver operating characteristic (AUROC) was used as an evaluation metric and 95% CI was calculated using the DeLong method^3^. The machine learning process was performed in R (v4.1.2) using Caret (v6.0-94) and recipes (v1.0.5) packages, and receiver operating characteristic (ROC) curve plots were made with pROC (v1.18.0). RF, NN and SVM-RBF models were made using respectively ranger v0.14.1, nnet v7.3-18 and kernlab v0.9-32 packages.

**Mapping incidence**

Incidence proportion map was made using packages rnaturalearth v0.3.2 and sf v1.0-12.

**Supplementary Table 1.** Incidence proportion for PI-IBS development in Dutch intercontinental travellers after traveller’s diarrhoea by sub region

|  | Number of travellers  At risk (n=539) | Number of travellers  who acquired PI-IBS  (n=65) | PI-IBS incidence  proportion (95% CI)ǂ |
| --- | --- | --- | --- |
| Central and Eastern Africa | 68 (12.6%) | 10 (15.4%) | 14.7 (6.9-18.4) |
| Northern Africa | 30 (5.6%) | 3 (4.6%) | 10.0 (3.8-17.6) |
| Southern Africa | 26 (4.8%) | 2 (3.1%) | 7.7 (3.0-16.5) |
| Western Africa | 36 (6.7%) | 3 (4.6%) | 8.3 (3.1-15.1) |
| Multiple sub regions in Africa | 15 (2.8%) | 3 (4.6%) | 20.0 (8.3-30.6) |
| Caribbean and Central America | 21 (3.9%) | 1 (1.5%) | 4.8 (2.4-15.5) |
| South America | 48 (8.9%) | 8 (12.3%) | 16.7 (7.3-20.9) |
| Multiple sub regions in America | 8 (1.5%) | 0 (0.0%) | 0.00 |
| Eastern Asia | 24 (4.5%) | 1 (1.5%) | 4.2 (1.9-13.8) |
| Southern Asia | 53 (9.8%) | 14 (21.5%) | 26.4 (12.2-27.5) |
| South eastern Asia | 176 (32.7%) | 19 (29.2%) | 10.8 (6.1-13.4) |
| Western Asia | 5 (0.9%) | 0 (0.0%) | 0.00 |
| Multiple sub regions in Asia | 19 (3.5%) | 1 (1.5%) | 5.3 (2.8-16.9) |
| Multiple regions | 10 (1.9%) | 0 (0.0%) | 0.00 |
| Total | 539 (100.0%) | 65 (100.0%) | 12.1 (8.3-13.9) |
| ǂBased on binomial distribution (Wilson’s score interval). | | | |

**Supplementary Table 2.** Univariable logistic regression analysis on potential predictors for PI-IBS acquisition among at risk travellers (n=539)

|  | **All travellers** **at risk**  (n=539)* | **Travellers who developed PI-IBS**  (n=65, n/N%)** | **OR**  **(95% CI)** | **P-value** | **Adjusted OR**  **(95% CI)** | **Adjusted**  **p-value** |
| --- | --- | --- | --- | --- | --- | --- |
| **Median age during arrival at destination** [IQR] | 52.44 [33.28:60.64] | 42.61 [27.43:57.14] | 0.97 (0.96-0.99) | <0.001 | 0.99 (0.96-1.02) | 0.38 |
| **Median BMI in kg/square meters [IQR]** | 24.69 [22.60:27.13] | 24.09 [22.61:26.15] | 0.95 (0.88-1.03) | 0.23 | 0.96 (0.87-1.06) | 0.44 |
| **Sex** |  |  |  |  |  |  |
| Male | 269 (49.9%) | 20/269 (7.4%) | 1 |  |  |  |
| Female | 270 (50.1%) | 45/270 (16.7%) | 2.49 (1.43-4.35) | 0.001 | 1.47 (0.71-3.04) | 0.30 |
| **Education level** |  |  |  |  |  |  |
| No education, elementary school or prevocational secondary education | 64 (11.9%) | 7/64 (10.9%) | 1 |  |  |  |
| Vocational secondary education, senior general secondary education or education up to university | 125 (23.2%) | 15/125 (12.0%) | 1.11 (0.42-2.88) | 0.83 |  |  |
| Higher professional education | 189 (35.1%) | 19/189 (10.1%) | 0.91 (0.36-2.28) | 0.84 |  |  |
| Academic (university) education | 161 (29.9%) | 24/161 (14.9%) | 1.43 (0.58-3.50) | 0.44 |  |  |
| **Drinking alcohol** |  |  |  |  |  |  |
| No | 42 (7.8%) | 8/42 (19.0%) | 1 |  |  |  |
| Yes | 497 (92.2%) | 57/497 (11.5%) | 0.55 (0.24-1.25) | 0.15 |  |  |
| **Smoking situation prior to travel** |  |  |  |  |  |  |
| No | 228 (42.5%) | 31/228 (13.6%) | 1 |  |  |  |
| Former smoker | 196 (36.6%) | 24/196 (12.2%) | 0.89 -1.57) | 0.68 | 1.17 (0.53-2.57) | 0.70 |
| Yes | 112 (20.9%) | 9/112 (8.0%) | 0.56 (0.26-1.21) | 0.14 | 0.28 (0.10-0.79) | 0.02 |
| **Region visited** |  |  |  |  |  |  |
| America | 77 (14.3%) | 9/77 (11.7%) | 1 |  |  |  |
| Africa | 175 (32.5%) | 21/175 (12.0%) | 1.03 (0.45-2.37) | 0.94 |  |  |
| Asia | 277 (51.4%) | 35/277 (12.6%) | 1.09 (0.50-2.39) | 0.82 |  |  |
| Multiple continents | 10 (1.9%) | 0/10 (0.0%) | 0 | 1 |  |  |
| **Reason for travel** |  |  |  |  |  |  |
| Vacation | 468 (86.8%) | 53/468 (11.3%) | 1 |  |  |  |
| Business/work or internship | 40 (7.4%) | 5/40 (12.5%) | 1.12 (0.42-2.98) | 0.82 |  |  |
| Other | 31 (5.8%) | 7/31 (22.6%) | 2.28 (0.94-5.56) | 0.07 |  |  |
| **Travel duration (days)** | 20 [15-26] | 21 [ 15-28] | 1.01 (1.00-1.03) | 0.17 | 0.99 (0.96-1.01) | 0.38 |
| **General health prior to travel** |  |  |  |  |  |  |
| Excellent | 155 (28.9%) | 11/155 (7.1%) | 1 |  |  |  |
| Good | 331 (61.8%) | 45/331 (13.6%) | 2.06 (1.03-4.10) | 0.04 | 0.88 (0.33-2.39) | 0.81 |
| Reasonable, mediocre or bad | 50 (9.3%) | 8/50 (16.0%) | 2.49 (0.94-6.60) | 0.07 | 0.772 (0.17-3.6) | 0.74 |
| **Depressive feelings up to three months prior to travel** |  |  |  |  |  |  |
| No | 470 (87.2%) | 55/470 (11.7%) | 1 |  |  |  |
| Yes | 69 (12.8%) | 10/69 (14.5%) | 1.30 (0.63-2.70) | 0.48 |  |  |
| **Antibiotics up to three months before travel** |  |  |  |  |  |  |
| No | 478 (89.0%) | 58/478 (12.1%) | 1 |  |  |  |
| Yes | 59 (11.0%) | 7/59 (11.9%) | 0.98 (0.42-2.25) | 0.95 |  |  |

| **Heart burn up to three months prior to travel** |  |  |  |  |  |  |
| --- | --- | --- | --- | --- | --- | --- |
| No | 427 (79.2%) | 46/427 (10.8%) | 1 |  |  |  |
| Yes | 112 (20.8%) | 19/112 (17.0%) | 1.69 (0.95-3.02) | 0.08 | 1.29 (0.58-2.89) | 0.54 |
| **Feeling tired up to three months prior to travel** |  |  |  |  |  |  |
| No | 314 (58.3%) | 23/314 (7.3%) | 1 |  |  |  |
| Yes | 225 (41.7%) | 42/225 (18.7%) | 2.90 (1.69-4.99) | <.001 | 1.79 (0.87-3.65) | 0.113 |
| **Stomach ache up to three months prior to travel** |  |  |  |  |  |  |
| No | 436 (80.9%) | 41/436 (9.4%) | 1 |  |  |  |
| Yes | 103 (19.1%) | 24/103 (23.3%) | 2.93 (1.67-5.12) | <.001 |  |  |
| **Stomach cramps up to three months prior to travel** |  |  |  |  |  |  |
| No | 447 (82.9%) | 42/447 (9.4%) | 1 |  |  |  |
| Yes | 92 (17.1%) | 23/92 (25.0%) | 3.21 (1.82-5.68) | <.001 | 1.28 (0.57-2.86) | 0.56 |
| **Vomiting in the past three months prior to travel** |  |  |  |  |  |  |
| No | 495 (91.8%) | 55/495 (11.1%) | 1 |  |  |  |
| Yes | 44 (8.2%) | 10/44 (22.7%) | 2.35 (1.10-5.03) | 0.03 | 2.46 (0.86-6.98) | 0.09 |
| **Nausea in the past three months prior to travel** |  |  |  |  |  |  |
| No | 457 (84.8%) | 46/457 (10.1%) | 1 |  |  |  |
| Yes | 82 (15.2%) | 19/82 (23.2%) | 2.70 (1.48-4.89) | 0.001 | 1.22 (0.51-2.90) | 0.66 |
| **Chronic illness** |  |  |  |  |  |  |
| No | 419 (78.3%) | 46/419 (11.0%) | 1 |  |  |  |
| Yes | 116 (21.7%) | 19/116 (16.4%) | 1.59 (0.89-2.84) | 0.12 | 2.35 (1.08-5.13) | 0.03 |
| **Diet** |  |  |  |  |  |  |
| No diet | 486 (90.2%) | 51/486 (10.5%) | 1 |  |  |  |
| Vegetarian or Vegan | 31 (5.8%) | 8/31 (25.8%) | 2.97 (1.26-6.98) | 0.01 | 3.49 (1.13-10.73) | 0.03 |
| Other diet (Jewish, Islamic or other) | 22 (4.1%) | 6/22 (27.3%) | 3.20 (1.20-8.54) | 0.02 | 5.69 (1.56-20.82) | 0.01 |
| **Probiotics use up till 12 months prior to travel** (capsules/tablets/in dairy) |  |  |  |  |  |  |
| No | 315 (59.0%) | 35/315 (11.1%) | 1 |  |  |  |
| Seldom | 113 (21.2%) | 16/113 (14.2%) | 1.32 (0.70-2.49) | 0.39 |  |  |
| Occasionally | 58 (10.9%) | 5/58 (8.6%) | 0.76 (0.28-2.02) | 0.57 |  |  |
| Often or daily | 48 (9.0%) | 8/48 (16.7%) | 1.60 (0.69-3.69) | 0.27 |  |  |
| **Anxiety up to three months prior to travel** |  |  |  |  |  |  |
| No | 505 (93.7%) | 57/505 (11.3%) | 1 |  |  |  |
| Yes | 34 (6.3%) | 8/34 (23.5%) | 2.42 (1.05-5.60) | 0.04 | 1.46 (0.44-4.85) | 0.54 |
| **Main accommodation during travel** |  |  |  |  |  |  |
| Multiple accommodations | 164 (30.4%) | 20/164 (12.2%) | 1 |  |  |  |
| Luxury stay (all-inclusive/4-5 stars) | 103 (19.1%) | 12/103 (11.7%) | 0.95 (0.44-2.04) | 0.89 |  |  |
| Hotel or apartment (0-3 stars) | 154 (28.6%) | 19/154 (12.3%) | 1.01 (0.52-1.98) | 0.97 |  |  |
| Low budget (guesthouse/hostel) | 67 (12.4%) | 10/67 (14.9%) | 1.26 (0.56-2.86) | 0.58 |  |  |
| Family/locals | 25 (4.6%) | 2/25 (8.0%) | 0.62 (0.14-2.86) | 0.55 |  |  |
| Tent. ship or other | 26 (4.8%) | 2/26 (7.7%) | 0.60 (0.13-2.73) | 0.51 |  |  |

| **Antibiotics use during and/or directly after travel** |  |  |  |  |  |  |
| --- | --- | --- | --- | --- | --- | --- |
| No | 486 (90.8%) | 54/486 (11.1%) | 1 |  |  |  |
| Yes | 49 (9.2%) | 10/49 (20.4%) | 2.05 (0.97-4.34) | 0.06 | 2.96 (1.14-7.66) | 0.03 |
| **General health during travel** |  |  |  |  |  |  |
| Excellent | 140 (26.2%) | 6/140 (4.3%) | 1 |  |  |  |
| Good | 343 (64.2%) | 49/343 (14.3%) | 3.72 (1.56-8.90) | 0.003 | 5.09 (1.59-16.33) | 0.01 |
| Reasonable, mediocre, or bad | 51 (9.6%) | 10/51 (19.6%) | 5.45 (1.87-15.89) | 0.002 | 5.48 (1.23-24.33) | 0.03 |
| **Anxiety during travel** |  |  |  |  |  |  |
| No | 513 (95.2%) | 59/513 (11.5%) | 1 |  |  |  |
| Yes | 26 (4.8%) | 6/26 (23.1%) | 2.31 (0.89-5.98) | 0.09 | 1.31 (0.35-4.95) | 0.69 |
| **Diarrhoea inhibitors during travel** |  |  |  |  |  |  |
| No | 336 (62.3%) | 38/336 (11.3%) | 1 |  |  |  |
| Yes | 203 (37.7%) | 27/203 (13.3%) | 1.20 (0.71-2.04) | 0.49 |  |  |
| **Antacids use during travel** |  |  |  |  |  |  |
| No | 510 (94.6%) | 55/510 (10.8%) | 1 |  |  |  |
| Yes | 29 (5.4%) | 10/29 (34.5%) | 4.35 (1.93-9.84) | <.001 | 1.69 (0.50-5.66) | 0.40 |
| **Feeling tired during travel** |  |  |  |  |  |  |
| No | 308 (57.1%) | 27/308 (8.8%) | 1 |  |  |  |
| Yes | 231 (42.9%) | 38/231 (16.5%) | 2.05 (1.21-3.47) | 0.01 | 0.66 (0.31-1.40) | 0.28 |

| **Heart burn during travel** |  |  |  |  |  |  |
| --- | --- | --- | --- | --- | --- | --- |
| No | 458 (85.0%) | 45/458 (9.8%) | 1 |  |  |  |
| Yes | 81 (15.0%) | 20/81 (24.7%) | 3.01 (1.67-5.44) | <.001 | 1.35 (0.56-3.23) | 0.50 |
| **Vomiting during travel** |  |  |  |  |  |  |
| No | 463 (85.9%) | 49/463 (10.6%) | 1 |  |  |  |
| Yes | 76 (14.1%) | 16/76 (21.1%) | 2.25 (1.21-4.21) | 0.01 |  |  |
| **Stomach ache during travel** |  |  |  |  |  |  |
| No | 314 (58.3%) | 20/314 (6.4%) | 1 |  |  |  |
| Yes | 225 (41.7%) | 45/225 (20.0%) | 3.68 (2.10-6.42) | <.001 |  |  |
| **Weight loss during travel** |  |  |  |  |  |  |
| No | 410 (76.1%) | 37/410 (9.0%) | 1 |  |  |  |
| Yes | 129 (23.9%) | 28/129 (21.7%) | 2.80 (1.63-4.79) | <.001 | 2.24 (1.07-4.7) | 0.03 |
| **Nausea during travel** |  |  |  |  |  |  |
| No | 350 (64.9%) | 21/350 (6.0%) | 1 |  |  |  |
| Yes | 189 (35.1%) | 44/189 (23.3%) | 4.75 (2.73-8.28) | <.001 | 2.25 (1.08-4.69) | 0.03 |
| **Stomach cramp during travel** |  |  |  |  |  |  |
| No | 209 (38.8%) | 7/209 (3.3%) | 1 |  |  |  |
| Yes | 330 (61.2%) | 58/330 (17.6%) | 6.15 (2.75-13.76) | <.001 | 5.92 (2.17-16.12) | <0.001 |
| **Obstipation during travel** |  |  |  |  |  |  |
| No | 445 (82.6%) | 44/445 (9.9%) | 1 |  |  |  |
| Yes | 94 (17.4%) | 21/94 (22.3%) | 2.62 (1.47-4.67) | 0.001 | 1.50 (0.69-3.29) | 0.31 |
| **Fever during travel** |  |  |  |  |  |  |
| No | 487 (90.4%) | 55/487 (11.3%) | 1 |  |  |  |
| Yes | 52 (9.6%) | 10/52 (19.2%) | 1.87 (0.89-3.94) | 0.10 |  |  |
| **Depressive feelings during travel** |  |  |  |  |  |  |
| No | 512 (95.0%) | 59/512 (11.5%) | 1 |  |  |  |
| Yes | 27 (5.0%) | 6/27 (22.2%) | 2.19 (0.85-5.66) | 0.10 |  |  |

* And ** numbers do not add up to 539 or 65 due to missing numbers. Missing values were assumed to be missing at random. The numerator represents the number of travellers who acquired PI-IBS after traveller’s diarrhoea (TD) and the denominator represent the number of travellers at risk. IQR, interquartile range.

**Supplementary Figure 1**. Overall receiver operating characteristic (ROC) curves from random forest, neural network, and support vector machine models. AUC = area under receiver operating characteristic

References

1. Hosmer Jr DW, Lemeshow S, Sturdivant RX. Applied logistic regression: John Wiley & Sons; 2013.

2. Mickey RM, Greenland S. The impact of confounder selection criteria on effect estimation. *American journal of epidemiology* 1989; **129**(1): 125-37.

3. DeLong ER, DeLong DM, Clarke-Pearson DL. Comparing the areas under two or more correlated receiver operating characteristic curves: a nonparametric approach. *Biometrics* 1988: 837-45.
